# Vedolizumab in biologic-naïve ulcerative colitis: a multicenter real-world evaluation of clinical benefits and exploratory inflammatory indices

**DOI:** 10.3389/fmed.2026.1897457

**Published:** 2026-07-17

**Authors:** Hao Li, Fanfan Zhu, Jingyi Yang, Xin Yao, Yunlong Sun, Wenbin Zeng, Dongping Lai, Shanneng Tang, Linyan Shen, Tao Zhang

**Affiliations:** 1 Guangxi University of Chinese Medicine, Nanning, China; 2 Department of Gastroenterology, Affiliated Wuxi Hospital of Nanjing University of Chinese Medicine, Wuxi, China; 3 Department of Gastroenterology, Guilin Hospital of Integrated Traditional Chinese and Western Medicine, Guilin, China; 4 Department of Gastroenterology, The Second Affiliated Hospital of Guangxi University of Chinese Medicine, Nanning, China

**Keywords:** biologic-naïve, real-world evidence, systemic immune-inflammation index, ulcerative colitis, vedolizumab

## Abstract

**Background and aims:**

Although vedolizumab (VDZ) serves as a primary biologic for ulcerative colitis (UC), long-term data in Chinese biologic-naïve cohorts and cost-effective prognostic biomarkers remain lacking. We evaluated the 54-week clinical benefits of VDZ as a first-line biologic in a Chinese cohort and investigated the clinical utility of systemic immune-inflammation indices (SII, NLR, PLR) in reflecting long-term outcomes and systemic inflammatory clearance during treatment de-escalation.

**Methods:**

This multicenter retrospective study enrolled biologic-naïve UC patients receiving VDZ. The primary endpoint was week-54 clinical remission, primarily evaluated using the full analysis set (FAS) with last observation carried forward (LOCF), accompanied by non-responder imputation (NRI) as a supplemental extreme-scenario analysis. Baseline indices were evaluated for predictive value, alongside an exploratory longitudinal analysis for patients undergoing early treatment de-escalation.

**Results:**

Among 41 patients in the FAS, the 54-week drug retention rate was 76.8%. Clinical remission was 73.2% (primary FAS-LOCF), with a conservative NRI estimate of 46.3%. In the paired endoscopy subgroup, 83.3% achieved endoscopic remission. While baseline SII and NLR correlated positively with C-reactive protein, they lacked robust predictive value for long-term remission. Instead, shorter disease duration was the sole independent predictor (OR = 0.84, *P* = 0.024). Notably, exploratory analysis revealed that declining SII trajectories coincided with successful treatment de-escalation. No unexpected safety signals emerged.

**Conclusions:**

Vedolizumab as a first-line biologic provides substantial clinical benefits and safety in bio-naive UC patients. While baseline inflammatory indices lack predictive power, their dynamic decline parallels clinical improvement. Due to the retrospective reliance on clinical symptoms and high rates of missing endoscopic data, these exploratory findings warrant cautious interpretation and prospective validation.

## 1 Introduction

Ulcerative colitis (UC) is a chronic inflammatory bowel disease with a significantly rising incidence in newly industrialized countries such as China (1–3). The efficacy of vedolizumab (VDZ) has been firmly established in pivotal clinical trials (4).

Historically, in Western real-world cohorts, vedolizumab was frequently utilized as a second-line treatment option (5). In China, infliximab and vedolizumab were approved almost simultaneously for ulcerative colitis around 2020, promoting initial vedolizumab selection. In parallel, recent research has shifted toward biologic-naïve cohorts (6, 7). Evaluating this unconfounded population clarifies true real-world outcomes while guiding personalized frontline management.

While massive neutrophil infiltration into the intestinal mucosa is a hallmark of UC, this localized pathology is intrinsically driven by the accelerated recruitment and mobilization of circulating immune cells (8). As IBD imposes a growing burden on global healthcare systems (9, 10), inexpensive and non-invasive indices derived from routine blood tests have gained widespread clinical attention (11, 12). For instance, Yan et al. (13) recently provided valuable insights into the potential of baseline SII in predicting VDZ clinical benefits. Building upon this foundational work, we established a multicenter cohort exclusively comprising biologic-naive UC patients to eliminate the potential influence of prior biologic exposure and evaluate the 54-week clinical benefits of VDZ. Within this unconfounded setting, we critically re-evaluated whether baseline blood markers could reliably predict clinical remission and exploratory endoscopic outcomes. Additionally, we conducted an exploratory analysis on a treatment de-escalation subgroup. By tracking dynamic shifts in these biomarkers, we sought to retrospectively evaluate their longitudinal variations during treatment transitions.

## 2 Methods

### 2.1 Patients

This retrospective study was conducted at three medical centers: the Second Affiliated Hospital of Guangxi University of Chinese Medicine, the Wuxi Affiliated Hospital of Nanjing University of Chinese Medicine, and the Guilin Integrated Traditional Chinese and Western Medicine Hospital. This study was approved by the Institutional Review Board of the Second Affiliated Hospital of Guangxi University of Chinese Medicine (Approval No.: KY-2026-49) and, thereafter, by all the other participating centers. The research complied with the Declaration of Helsinki. Given the retrospective nature of the study, the requirement for informed consent was waived, and all data were anonymized. The study design and reporting followed the STROBE statement guidelines (14).

Eligible patients were adults (≥18 years) with a confirmed UC diagnosis according to national guidelines (15). All participants initiated intravenous vedolizumab and provided complete baseline clinical and endoscopic data. We required at least one infusion and a subsequent clinical assessment for inclusion. We excluded patients with a history of total colectomy, though one patient with prior surgery was retained for safety analysis. Other exclusion criteria included prior vedolizumab exposure at other institutions (resulting in unavailable baseline data), complete loss to follow-up, and active malignancy.

**Figure 1:**
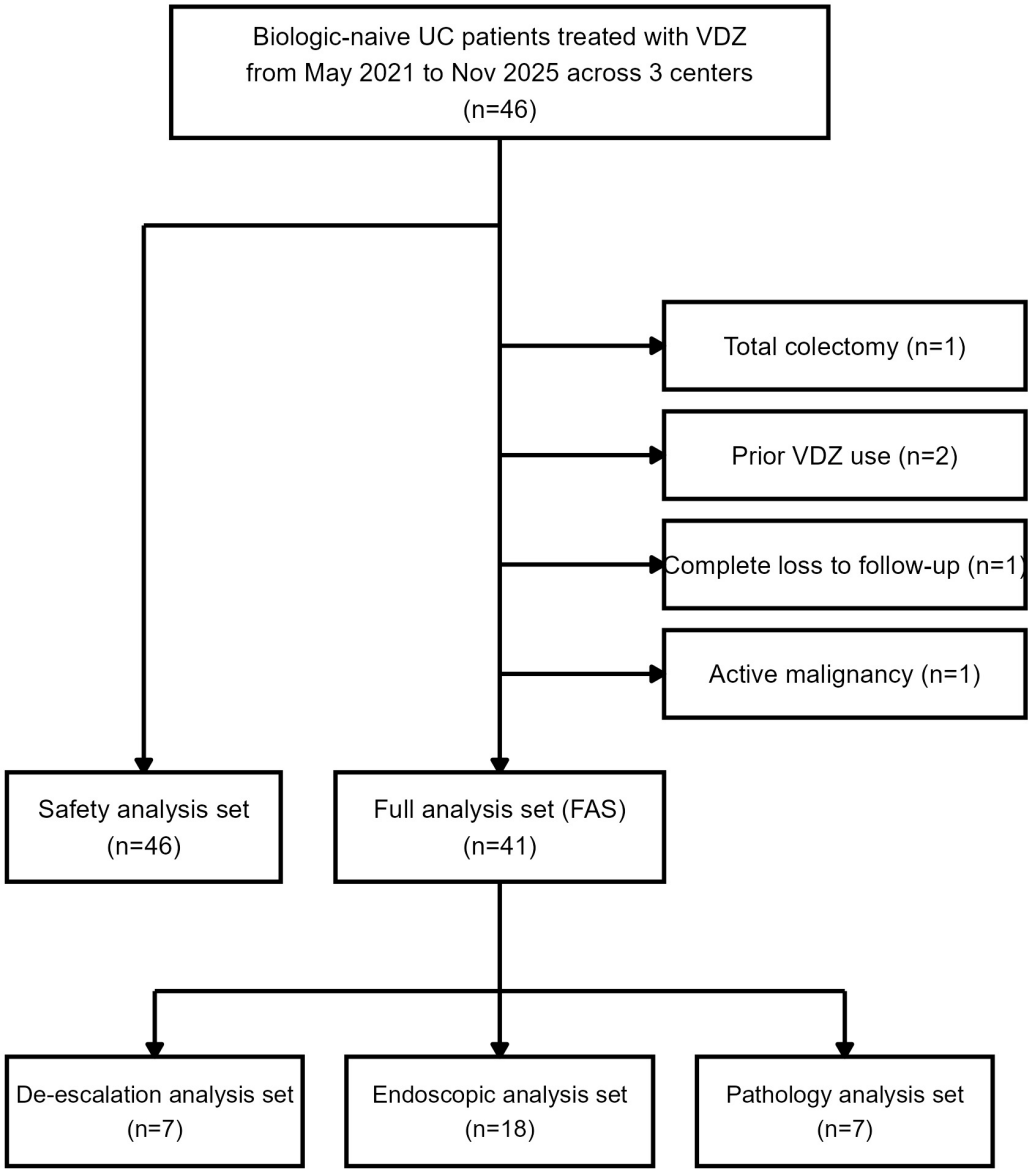
Flow diagram of patient selection and cohort definition.

Of note, we included three patients with latent tuberculosis after they completed prophylactic therapy. Overall, 46 screened patients received at least one VDZ infusion, constituting the safety analysis set. After excluding five ineligible cases, 41 patients formed the full analysis set (FAS). To address diverse clinical and biological endpoints, we established three exploratory analysis sets: the endoscopic analysis set (*n* = 18) and histological analysis set (*n* = 7) based on the availability of paired evaluations, and the de-escalation analysis set (*n* = 7) comprising patients who transitioned to conventional therapy following clinical improvement. Figure 1 detailed the participant disposition.

### 2.2 Treatment regimen

All patients received the standard intravenous vedolizumab (VDZ) regimen: 300 mg induction doses at Weeks 0, 2, and 6, followed by 300 mg maintenance doses every 8 weeks starting from week 14 until week 54 or treatment cessation. Stable doses of 5-aminosalicylates, corticosteroids (prednisone equivalent ≤ 20 mg/d), and immunosuppressants (azathioprine or 6-mercaptopurine) were permitted. Corticosteroid tapering was performed after the induction phase according to clinical response.

### 2.3 Data collection

Baseline data were collected within 14 days prior to the initial VDZ infusion. Follow-up visits were scheduled at weeks 14 and 30 (±2-week window) and week 54 (weeks 46–62). To minimize information bias, three trained independent researchers extracted and cross-checked data from electronic medical record systems, with discrepancies resolved by a senior clinician.

Extracted parameters included demographics, disease duration, treatment history, concomitant medications, partial Mayo score, Mayo endoscopic subscore (MES), C-reactive protein (CRP), hemoglobin (Hb), albumin (Alb), erythrocyte sedimentation rate (ESR), and white blood cell (WBC) count. Corticosteroid dosage and adverse events were recorded at each visit.

### 2.4 Clinical and endoscopic assessment

Patients achieved clinical remission if their partial Mayo score was ≤ 2 with a concurrent rectal bleeding subscore of 0. This aligns with STRIDE-II targets (16) and established real-world observations (17, 18). Clinical response required a partial Mayo score decrease of ≥2 points and 25% from baseline, alongside a rectal bleeding subscore reduction of ≥1 point (or an absolute score of 0 or 1). Endoscopic remission corresponded to a Mayo Endoscopic Subscore (MES) ≤ 1. A decrease of ≥1 point in MES from baseline constituted an endoscopic response. A Nancy Index ≤ 1 indicated histological remission. Corticosteroid-free clinical remission applied to baseline users who reached clinical remission at week 54 without any ongoing corticosteroid therapy.

### 2.5 Outcomes

The primary endpoint was the clinical remission rate at week 54. Secondary endpoints included clinical response, drug persistence, endoscopic remission, and histological improvement. Safety outcomes focused on serious adverse events (SAEs), adverse events leading to treatment discontinuation, infusion reactions, and infection events. Given the retrospective design, minor adverse events may not have been comprehensively recorded.

### 2.6 Statistical analysis

Data summaries utilized medians with interquartile ranges (IQR) for continuous variables and frequencies with percentages for categorical ones. The Mann–Whitney *U*-test or Fisher's exact test evaluated group differences. Spearman's rank method determined correlations, while the Wilcoxon signed-rank test assessed longitudinal shifts.

The week 54 clinical remission rate acted as the primary clinical endpoint. The last observation carried forward (LOCF) method imputed missing data within the full analysis set (FAS). Paired laboratory and endoscopic data underwent an observed-cases analysis. To evaluate the impact of missing data and establish strict clinical bounds, supplemental extreme-scenario analyses were performed using non-responder imputation (NRI) and baseline observation carried forward (BOCF).

Binary logistic regression identified predictors for 54-week clinical remission, yielding odds ratios (OR) and 95% confidence intervals (CI). We natural log-transformed skewed continuous variables (SII, NLR, PLR) before univariate analysis. Strict statistical protocols prevented the construction of multivariate models or receiver operating characteristic (ROC) curves for variables lacking univariate significance.

For patients (*n* = 7) undergoing treatment de-escalation after achieving clinical-endoscopic remission, we performed a descriptive longitudinal analysis. Spaghetti plots visualized the dynamic trajectories of markers from baseline to drug withdrawal. Due to the limited sample size, we deliberately omitted inferential statistical tests. All statistical operations utilized R software version 4.4.1 (R Foundation for Statistical Computing, Vienna, Austria), defining significance at a two-sided *P*-value < 0.05.

## 3 Results

### 3.1 Baseline characteristics and patient disposition

The full analysis set (FAS) comprised 41 bio-naive patients with ulcerative colitis (UC) receiving first-line vedolizumab (VDZ) therapy. Detailed baseline characteristics are summarized in Table 1. The median age of the cohort was 47.0 years (IQR: 32.5–58.5), with 51.2% being male. The median disease duration was 44.0 months, and extensive colitis (E3) was the most prevalent phenotype (53.7%). At baseline, the median partial Mayo score was 5.0 (IQR: 3.0–7.0), indicating a cohort with moderate-to-severe clinical activity. Additionally, 43.9% (18/41) of patients were receiving concomitant systemic corticosteroids at baseline.

**Table 1:** Baseline characteristics of biologic-naive patients with ulcerative colitis according to clinical remission.

Characteristics	Total (*N* = 41)	Remission (*n* = 30)	Non-remission (*n* = 11)
Sex, *n* (%)			
Male	21 (51.2)	16 (53.3)	5 (45.5)
Female	20 (48.8)	14 (46.7)	6 (54.5)
Age, years, median (IQR)	47.0 (32.5–58.5)	41.0 (32.5–56.8)	53.0 (50.0–60.5)
Disease duration, months, median (IQR)	44.0 (8.0–96.0)	34.5 (6.5–70.5)	63.0 (47.0–162.0)
Extent of disease, *n* (%)			
E1	5 (12.2)	5 (16.7)	0 (0)
E2	14 (34.1)	10 (33.3)	4 (36.4)
E3	22 (53.7)	15 (50.0)	7 (63.6)
Partial Mayo score, median (IQR)	5.0 (3.0–7.0)	5.0 (3.0–6.0)	7.0 (5.0–7.5)
MES,^a^ median (IQR)	3.0 (2.0–3.0)	2.0 (2.0–3.0)	3.0 (3.0–3.0)
Concomitant medications, *n* (%)			
Corticosteroids	18 (43.9)	11 (36.7)	7 (63.6)
Immunosuppressants	3 (7.3)	2 (6.7)	1 (9.1)
CRP, mg/L,^b^ median (IQR)	5.0 (3.5–6.8)	5.0 (2.7–5.0)	4.8 (3.9–18.1)
Hemoglobin, g/L, median (IQR)	127.0 (120.0–135.0)	127.5 (121.3–136.3)	112.0 (101.5–122.0)
NLR, median (IQR)	2.51 (1.84–3.24)	2.46 (1.83–3.25)	2.64 (2.09–3.13)
PLR, median (IQR)	169.35 (149.35–205.66)	166.88 (145.94–206.87)	170.77 (162.89–187.31)
SII, median (IQR)	674.20 (528.22–1061.38)	686.25 (486.51–1050.87)	674.20 (643.67–1037.78)

CRP, C-reactive protein; IQR, interquartile range; MES, Mayo endoscopic subscore; NLR, neutrophil-to-lymphocyte ratio; PLR, platelet-to-lymphocyte ratio; SII, systemic immune-inflammation index.

^a^Data available for 34 patients (remission, *n* = 27; non-remission, *n* = 7).

^b^Data available for 39 patients (remission, *n* = 28; non-remission, *n* = 11).

**Figure 2:**
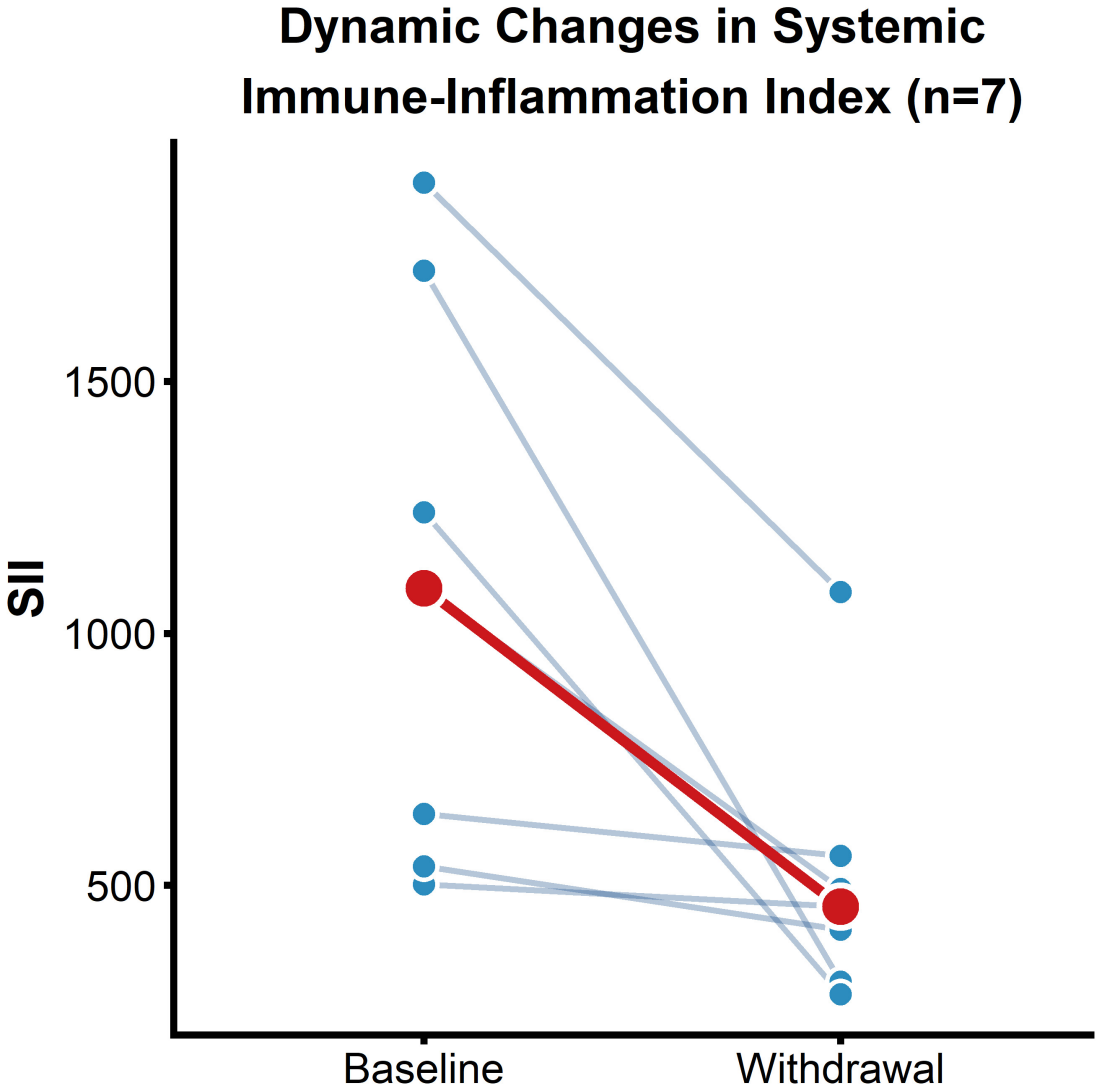
Exploratory longitudinal trajectories of the systemic immune-inflammation index (SII) in patients undergoing treatment de-escalation. Individual paired SII values (*n* = 7) are presented. The plot illustrates the absolute decrease in peripheral blood SII levels for each patient from baseline to the point of de-escalation in this exploratory subgroup.

Of the 41 patients in the FAS, 28 (68.3%) completed the 54-week follow-up. Among the 13 patients who did not complete the full 54-week follow-up, four remained on treatment but had not yet reached the week 54 assessment node at the study cutoff, while two discontinued therapy due to insufficient clinical response. The remaining seven patients underwent treatment de-escalation (transitioning to conventional non-biologic maintenance therapy). Notably, at the time of de-escalation, these patients had achieved clinical-endoscopic remission, characterized by a Modified Mayo Score ≤ 2 and a Mayo Endoscopic Subscore ≤ 1. This treatment transition was primarily associated with socio-economic factors and patient preference. Detailed clinical profiles and individual rationales for this subgroup are summarized in Supplementary Table S1. Retrospectively, an exploratory trajectory analysis for this de-escalation subgroup revealed a consistent downward trend in peripheral blood SII levels from baseline to the point of de-escalation (Figure 2). Terminal assessment data for all 13 patients were incorporated into the primary clinical analysis using the LOCF method.

### 3.2 Drug persistence and survival analysis

**Figure 3:**
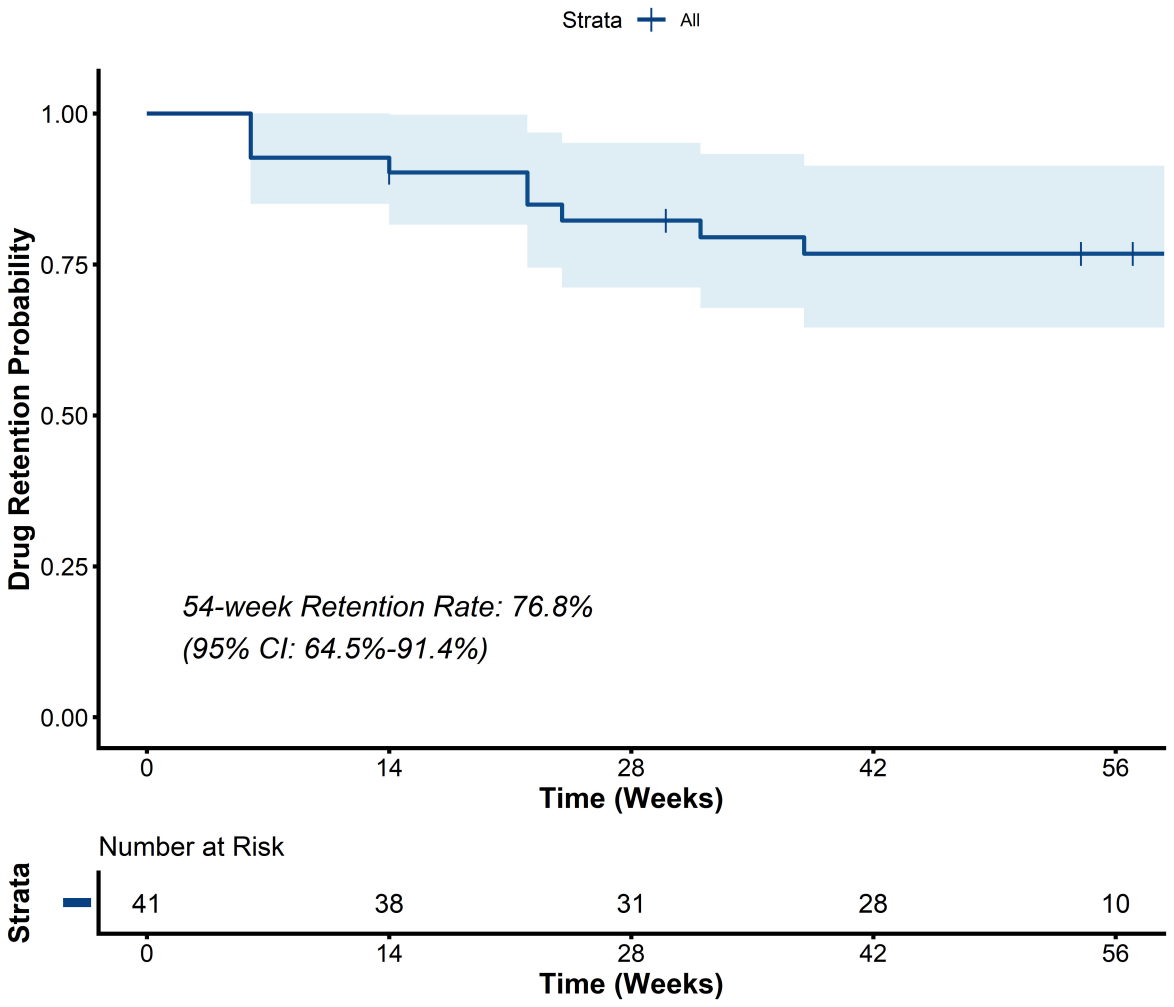
Kaplan–Meier curve of vedolizumab drug retention over 54 weeks. The cumulative drug retention probability at week 54 was 76.8% (95% CI: 64.5%−91.4%). The solid line represents the estimated retention probability, and the shaded area indicates the 95% CI. Vertical ticks denote censored data points. The number of patients at risk is displayed below the *x*-axis at 14-week intervals. CI, confidence interval.

The survival curve (Figure 3) showed a gradual decline in drug retention, with most discontinuations occurring within the initial 30 weeks. Although the primary retention analysis focused on the 54-week endpoint, the maximum follow-up period extended up to 118 weeks. Throughout this entire period, only five patients definitively switched to alternative biologics due to insufficient clinical response or adverse events (two cases within 54 weeks and three cases thereafter). Notably, the remaining patients who discontinued VDZ therapy predominantly did so after achieving early clinical improvement, undergoing planned treatment de-escalation to conventional maintenance therapy (*n* = 7).

### 3.3 Clinical benefits and laboratory improvements at week 54

Analysis of the full analysis set (FAS, *n* = 41) employed the LOCF approach. At week 54, the clinical remission rate was 73.2% (95% CI: 58.1%−84.3%) and the clinical response rate reached 70.7% (95% CI: 55.5%−82.4%). The median partial Mayo score significantly improved from 5.0 (IQR: 4.0) at baseline to 2.0 (IQR: 3.0) at week 54 (*P* < 0.001). Clinical remission (73.2%, 30/41) exceeded response (70.7%, 29/41) because patients with mild baseline scores achieved the absolute remission threshold ( ≤ 2) without the ≥2-point drop required for response.

Changes in laboratory parameters are detailed in Table 2. After 54 weeks of VDZ therapy, erythrocyte sedimentation rate (ESR) and white blood cell (WBC) counts decreased significantly (*P* = 0.027 and *P* = 0.011, respectively), and hemoglobin (Hb) levels significantly improved (*P* = 0.032). Changes in C-reactive protein (CRP) and serum albumin (Alb) did not reach statistical significance (*P* > 0.05). Notably, among the 18 patients receiving concomitant systemic corticosteroids at baseline, 88.9% (16/18) successfully discontinued corticosteroids by week 54, and 61.1% (11/18) achieved strict corticosteroid-free clinical remission. Only two patients required ongoing corticosteroid maintenance.

**Table 2:** Changes in laboratory parameters from baseline to week 54.

Parameter	*N*	Baseline (median, IQR)	Week 54 (median, IQR)	Median difference (95% CI)	*P* value
CRP (mg/L)	39	5.00 (1.90)	5.00 (3.95)	1.33 (−2.42, 6.65)	0.226
ESR (mm/h)	39	20.0 (23.5)	15.0 (22.0)	7.00 (1.00, 12.00)	0.027
WBC ( × 10^9^/L)	41	6.87 (2.09)	5.69 (1.87)	0.83 (0.15, 1.40)	0.011
Hb (g/L)	41	127 (20)	129 (26)	−5.00 (−10.00, −0.50)	0.032
Alb (g/L)	41	39.0 (7.7)	39.9 (5.1)	−0.95 (−2.45, 0.30)	0.147

Alb, albumin; CI, confidence interval; CRP, C-reactive protein; ESR, erythrocyte sedimentation rate; Hb, hemoglobin; IQR, interquartile range; WBC, white blood cell. Data are presented for the overall cohort (*n* = 41), with the exception of CRP and ESR (*n* = 39) due to missing laboratory values.

### 3.4 Endoscopic and histological assessment

Endoscopic and histological evaluations were restricted to patients with paired baseline and week 54 assessments (*n* = 18 for endoscopy and *n* = 7 for histology). Given the potential for selection bias, these findings are presented as exploratory results.

**Figure 4:**
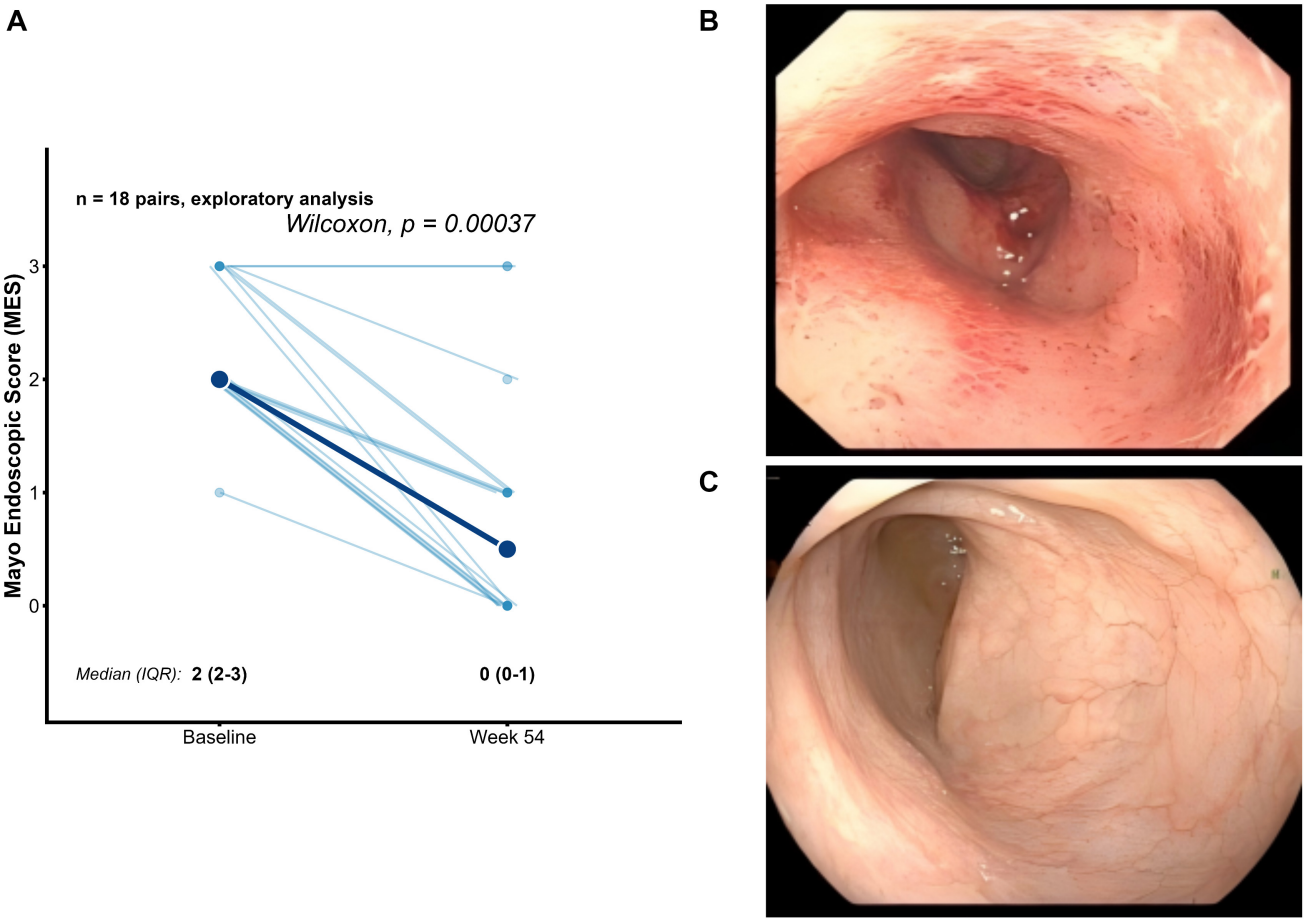
Endoscopic outcomes and representative mucosal healing at week 54. **(A)** Changes in the Mayo Endoscopic Score (MES) from baseline to week 54 in the paired subgroup (*n* = 18). Light blue lines represent individual trajectories, and the dark blue line indicates the median change. Statistical significance was determined using the Wilcoxon signed-rank test. **(B, C)** Representative colonoscopy images showing the same anatomical site (sigmoid colon) at baseline **(B)** and after 54 weeks of vedolizumab treatment **(C)**. MES, mayo endoscopic score.

In the endoscopic subgroup (*n* = 18), the median Mayo endoscopic subscore (MES) decreased significantly from 2.0 (IQR: 1.0) at baseline to 0.5 (IQR: 1.0) at week 54 (*P* < 0.001; Figure 4A). The endoscopic remission rate (MES ≤ 1) was 83.3% (15/18), and the endoscopic response rate (MES reduction ≥1) was 88.9% (16/18). Representative endoscopic images are provided in Figures 4B, C. Under the conservative non-responder imputation (NRI) analysis, treating all FAS patients (*n* = 41) with missing endoscopic data as failures, the week 54 endoscopic remission rate was 36.6% (15/41).

Among the 7 patients with paired colonic biopsies, histological activity was assessed using the Nancy Histological Index (NHI). The median NHI score remained 2.0 at both baseline and week 54 (*P* = 0.813). However, qualitative improvements, such as the restoration of crypt architecture and the resolution of inflammatory cell infiltration, were observed in several responders (Supplementary Figure S1).

### 3.5 Supplemental extreme-scenario analyses

**Figure 5:**
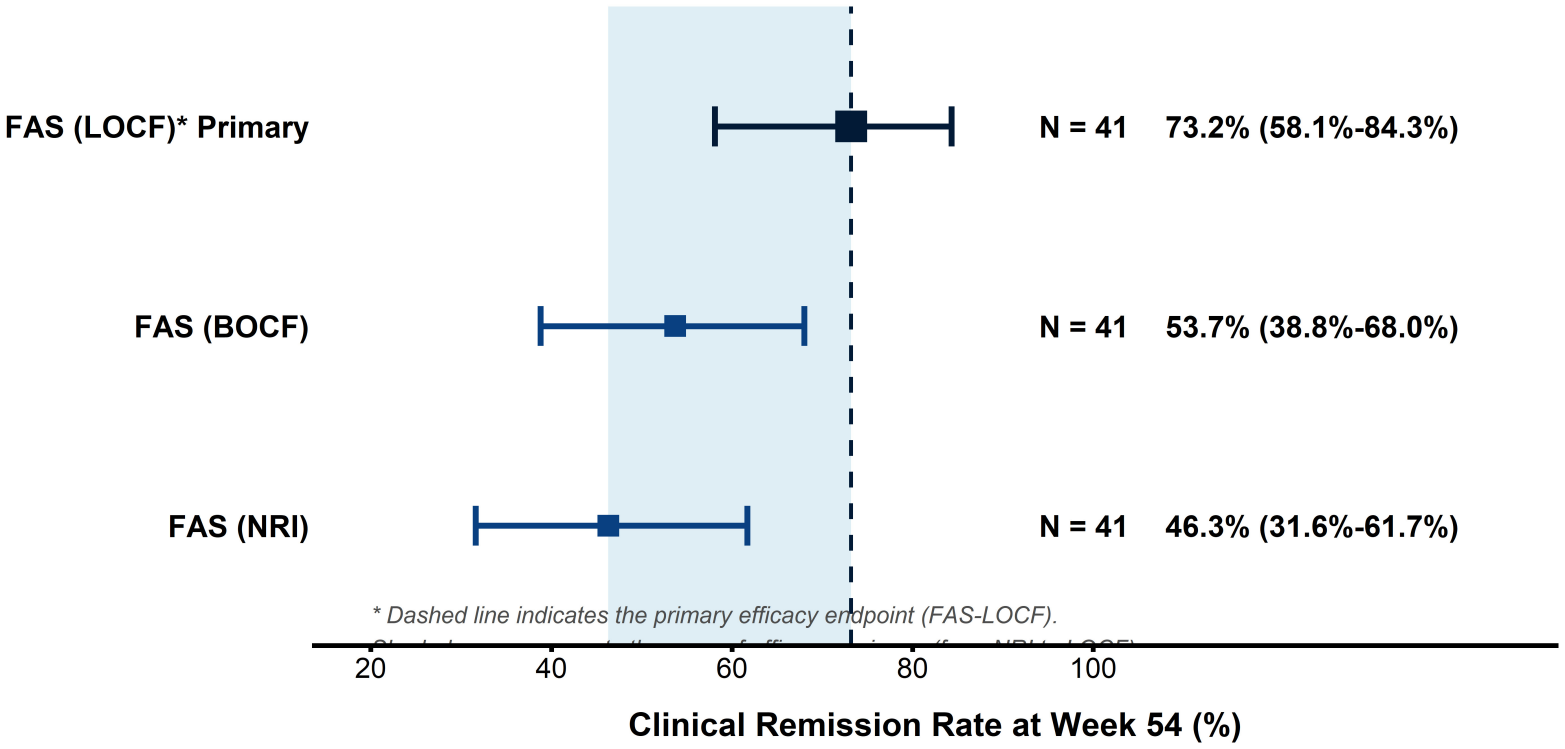
Supplemental extreme-scenario analysis of clinical remission rates at week 54. Forest plot comparing the primary efficacy analysis using LOCF with conservative imputation strategies (BOCF and NRI) in the full analysis set (*n* = 41). Squares indicate point estimates for remission rates, and horizontal error bars represent 95% confidence intervals. The vertical dashed line aligns with the primary LOCF estimate. The shaded area highlights the variance range between the most conservative estimate (NRI) and the primary analysis (LOCF). FAS, full analysis set; LOCF, last observation carried forward; BOCF, baseline observation carried forward; NRI, non-responder imputation.

To evaluate the influence of missing data handling on the primary endpoint, supplemental extreme-scenario analyses were conducted (Figure 5). The clinical remission rate was 46.3% (19/41, 95% CI: 31.6%−61.7%) using the NRI approach and 53.7% (22/41, 95% CI: 38.8%−68.0%) using the BOCF method, compared to the primary FAS-LOCF result of 73.2% (30/41, 95% CI: 58.1%−84.3%).

### 3.6 Subgroup analysis

Week 54 clinical remission rates (FAS-LOCF) stratified by baseline characteristics are detailed in Table 3. The clinical remission rate was 78.4% (29/37) in patients with a baseline partial Mayo score < 8 (*n* = 37) and 25.0% (1/4) in those with a score ≥8, demonstrating borderline significance (*P* = 0.052). Concomitant use of systemic corticosteroids or immunosuppressants at baseline did not significantly affect the week 54 clinical remission rates (*P* > 0.05). Given the substantial disparities in subgroup sample sizes, these findings are presented as exploratory observations, and no adjustments for multiple comparisons were applied.

**Table 3:** Subgroup analysis of clinical remission at week 54 (FAS, LOCF).

Subgroup	Clinical remission, % (*n*/*N*)	*P* value
Baseline partial Mayo score ≥8 (*n* = 4)	25% (1/4)	0.052
Baseline partial Mayo score < 8 (*n* = 37)	78.4% (29/37)	
Concomitant corticosteroids (*n* = 18)	61.1% (11/18)	0.164
No concomitant corticosteroids (*n* = 23)	82.6% (19/23)	
Concomitant immunosuppressants (*n* = 3)	66.7% (2/3)	1.000
No concomitant immunosuppressants (*n* = 38)	73.7% (28/38)	

FAS, full analysis set; LOCF, last observation carried forward. *P* values were calculated using Fisher's exact test.

### 3.7 Correlation of novel inflammatory indices with disease activity and their predictive value for 54-week clinical outcomes

**Figure 6:**
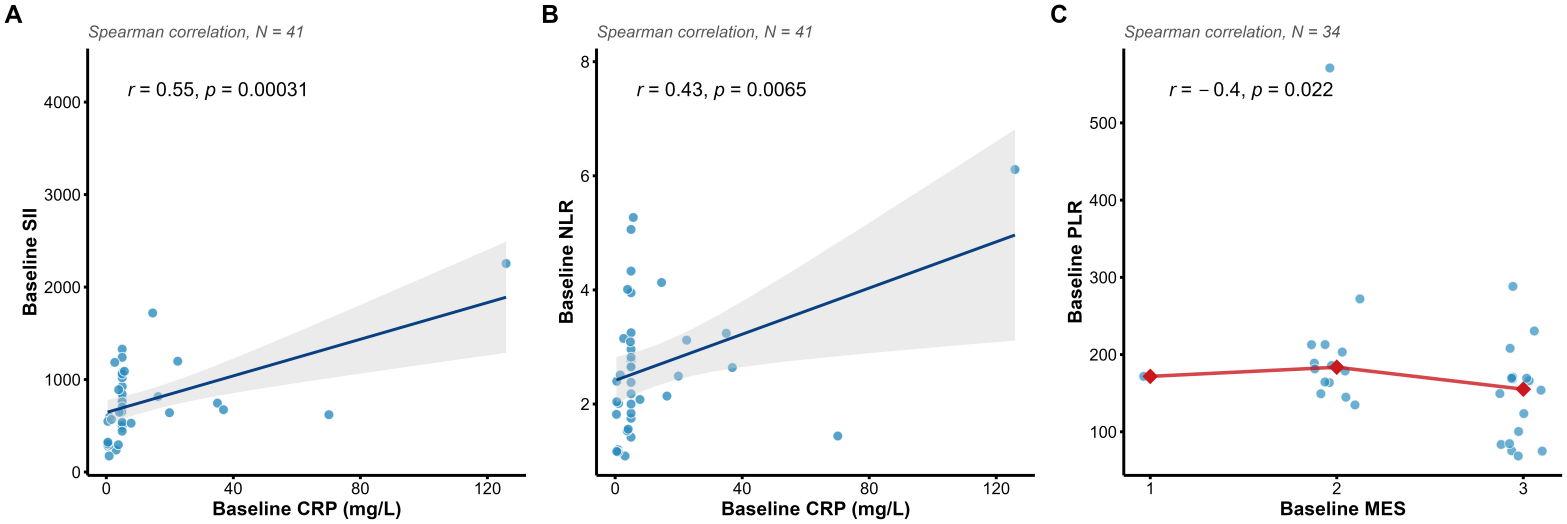
Correlations between baseline peripheral blood-derived inflammatory indices, C-reactive protein, and endoscopic activity. **(A, B)** Scatter plots of baseline C-reactive protein (CRP) against **(A)** systemic immune-inflammation index (SII) and **(B)** neutrophil-to-lymphocyte ratio (NLR) in the overall cohort (*n* = 41). Solid blue lines indicate the linear regression fit, and shaded areas represent 95% confidence intervals. **(C)** Scatter plot of baseline platelet-to-lymphocyte ratio (PLR) against the mayo endoscopic score (MES; *n* = 34). Points are horizontally jittered. Red diamonds connected by a solid line indicate the median PLR for each MES category. Statistical analyses were performed using Spearman's rank correlation. *r*, Spearman's correlation coefficient; CRP, C-reactive protein; MES, mayo endoscopic score; SII, systemic immune-inflammation index; NLR, neutrophil-to-lymphocyte ratio; PLR, platelet-to-lymphocyte ratio.

Spearman rank correlation analysis was initially performed to examine the relationships between baseline peripheral blood-derived inflammatory indices (SII, NLR, and PLR), disease activity, and conventional markers such as CRP (Figure 6). Baseline SII and NLR were significantly positively correlated with CRP (SII: *r* = 0.55, *P* < 0.001; NLR: *r* = 0.43, *P* = 0.006). Conversely, baseline PLR showed a negative correlation with endoscopic severity as measured by MES (*r* = −0.40, *P* = 0.022).

Univariate logistic regression analysis was performed to evaluate baseline predictors of 54-week clinical remission (Table 4). Systemic inflammatory indices were natural log-transformed due to right-skewness. These log-transformed indices, including Ln (SII), Ln (NLR), and Ln (PLR), showed no significant predictive value for long-term clinical remission (all *P* > 0.05). Baseline MES also demonstrated no significant predictive value, with its evaluation limited by complete separation. Shorter disease duration (per year) was the sole baseline factor significantly associated with 54-week clinical remission (OR = 0.84, 95% CI: 0.70–0.97; *P* = 0.024). Because no significant independent predictors were identified in the univariate analysis, multivariate regression and receiver operating characteristic (ROC) curve analyses were not performed.

**Table 4:** Univariate logistic regression analysis of factors associated with 54-week clinical remission.

Variables	Odds ratio (OR)	95% CI	*P*-value
Demographics and disease characteristics			
Age (per 10 years)	0.66	0.39–1.05	0.095
Gender (male vs. female)	1.20	0.30–5.00	0.797
Disease duration (per year)	0.84	0.70–0.97	**0.024**
Clinical and endoscopic activity			
Baseline MES	NA	NA	0.995
Laboratory parameters			
C-reactive protein (mg/L)	1.00	0.97–1.04	0.991
Erythrocyte sedimentation rate (mm/h)	0.97	0.94–1.01	0.144
Hemoglobin (g/L)	1.03	0.99–1.07	0.139
Albumin (g/L)	1.08	0.94–1.24	0.293
Systemic inflammatory indices (log-transformed)			
Ln (SII)	0.77	0.24–2.33	0.637
Ln (NLR)	0.84	0.18–3.87	0.814
Ln (PLR)	0.67	0.12–3.52	0.632

Bold *P*-values indicate statistical significance (*P* < 0.05).

CI, confidence interval; CRP, C-reactive protein; ESR, erythrocyte sedimentation rate; Ln, natural logarithm; MES, Mayo endoscopic subscore; NA, not applicable; NLR, neutrophil-to-lymphocyte ratio; PLR, platelet-to-lymphocyte ratio; SII, systemic immune-inflammation index.

The OR and CI for MES are presented as NA due to complete separation driven by the sample distribution in the single-variable model.

### 3.8 Safety analysis

Safety data over the entire observation period follow-up were evaluated in the safety analysis set (*n* = 46). Two serious adverse events (SAEs) were recorded, representing an overall incidence of 4.3% (2/46). All SAEs manifested as opportunistic intestinal infections. Specifically, cytomegalovirus (CMV) colitis was confirmed in these two patients, with one case complicated by a concurrent Clostridioides difficile infection (CDI). One infection presented early, prompting exclusion from the FAS. The second emerged after the 54-week evaluation. VDZ therapy was immediately and permanently discontinued in all affected individuals. Following standard antiviral and anti-CDI treatments, both patients achieved clinical resolution and subsequently switched to infliximab maintenance therapy. No severe infusion reactions, incident malignancies, or deaths were observed in the cohort.

## 4 Discussion

This multicenter study demonstrates the long-term clinical benefits and safety of vedolizumab in biologic-naïve patients with ulcerative colitis (UC) in China. Over the 54-week follow-up, patients achieved stable clinical remission (73.2%) and high drug persistence (76.8%). Conservative supplemental extreme-scenario analyses were also performed using non-responder imputation (NRI) and baseline observation carried forward (BOCF) frameworks. Furthermore, this study evaluated the potential of systemic immune-inflammation indices (SII, NLR, and PLR) for predicting long-term clinical outcomes and monitoring treatment transitions. Though improvements were noted in the endoscopic (*n* = 18) and histological (*n* = 7) subgroups, these findings remain exploratory due to the retrospective design and limited sample size. Aligning with recent domestic real-world evidence (7), our findings provide an objective reference for the individualized clinical management of frontline VDZ therapy.

### 4.1 Clinical efficacy and the bio-naive advantage

The week 54 clinical remission rate observed via FAS-LOCF (73.2%) was numerically higher than the 40%−60% range reported in previous real-world studies (19, 20). This discrepancy arises primarily from differences in statistical strategies and population characteristics. While FAS-LOCF evaluates the therapeutic potential in patients maintaining treatment, the application of stringent NRI provides a conservative estimate analogous to the intention-to-treat analysis in clinical trials. These complementary analytical approaches define the spectrum of therapeutic benefits; notably, the NRI remission rate of 46.3% in this study is comparable to the TNF-naive subgroup in the *post-hoc* analysis of GEMINI 1 (46.9%) (21) and the ERELATE study (19), ensuring the reliability and cross-study comparability of these outcomes.

The completely biologic naive cohort likely drives the favorable outcomes and high drug persistence (76.8%). Extensive evidence suggests that bio-naive patients achieve significantly better outcomes with VDZ than bio-experienced patients (21–23); this advantage is corroborated by the cohort study of Kopylov et al. (24) and clinical trial data for filgotinib (the SELECTION trial) (25). Compared to cohorts reported by Kobayashi et al. (20) and Amiot et al. (26) that included high proportions of bio-experienced patients, the favorable drug persistence in this study suggests that initiating VDZ during the bio-naive stage may yield more stable long-term maintenance effects.

Potential benefits in the biologic-naïve population were further suggested by exploratory endoscopic outcomes in a subset of patients. Although the paired endoscopic subgroup (*n* = 18) reached a remission rate of 83.3%, the NRI analysis (36.6%) provides a more rigorous reference. Despite inter-study heterogeneity, a horizontal comparison suggests a potential increasing trend in endoscopic remission alongside the rising proportion of bio-naive patients across different cohorts: VIOLET (62.2% remission; 70.7% naïve) (27), Huang et al. (70.6% remission; 84.1% naïve) (17), and the present study (83.3% remission; 100% naïve).

Furthermore, histological outcomes were explored using the Nancy index, in accordance with the STRIDE-II consensus (16). Although the small sample size (*n* = 7) limited statistical power (*P* = 0.813), incorporating histological assessment reflects a clinical shift toward achieving deep healing. Collectively, these findings reinforce the clinical value of VDZ as a first-line biologic. Since bio-experienced patients often face efficacy attenuation during subsequent therapies (22, 28), prioritizing VDZ in eligible patients may offer a more favorable long-term prognosis than switching biologics after prior failure.

### 4.2 Missing data handling and supplemental extreme-scenario analysis

Real-world data inherently face attrition bias driven by non-failure events. We implemented alternative evaluation strategies including BOCF, alongside the NRI method (29). Consistent with the conservative reporting standards adopted in large real-world cohorts (28), we applied the strict NRI approach to define a conservative lower boundary, treating all patients without 54-week data as treatment failures (30). However, we must explicitly acknowledge the clinical and statistical limitations of these mathematical imputations. As ulcerative colitis is a dynamically changing disease, applying BOCF relies on the unrealistic assumption that a patient's condition remains static from baseline over 54 weeks. Furthermore, these statistical methods cannot fully compensate for the constraints of a limited sample size, low statistical power, and the lack of comprehensive objective measurements. Ultimately, they cannot eliminate the selection bias inherent to high missing data rates and may produce estimates that underestimate the true statistical uncertainty of the results. Nevertheless, by contrasting the primary LOCF analysis, which prevents the artificial deflation of therapeutic estimates caused by strictly penalizing non-failure attrition, with the conservative NRI boundaries, these complementary analyses define a pragmatic therapeutic range of 46.3%−73.2%. Rather than pursuing an idealized statistical deduction, this approach honestly reflects the objective constraints of real-world clinical follow-up, thereby providing a more pragmatic reference for clinical practice.

### 4.3 Predictors of clinical benefits and safety

In this study, baseline PLR negatively correlated with MES. This result differs from findings in severe UC cohorts (12, 31). This variation might be attributed to the non-significant elevation of inflammatory markers like CRP in our cohort; indeed, up to half of patients with endoscopically active UC exhibit normal CRP levels (32). In such settings of low systemic inflammatory burden, PLR lacks discriminatory capacity (33). However, this remains a preliminary exploration. Given the limited sample size of our retrospective cohort, we cannot exclude the influence of unknown residual confounding. Baseline SII, NLR, and PLR did not predict clinical outcomes at Week 54. This lack of predictive ability may be due to the gut-selective mechanism of vedolizumab. Vedolizumab is a gut-selective integrin antagonist. It primarily inhibits lymphocyte homing to the intestinal mucosa (4). It does not directly target the systemic load of inflammatory cells in peripheral blood. In contrast, NLR and SII perform better at predicting the clinical benefits of systemic anti-TNF agents (34). Furthermore, our cohort was purely bio-naive with mild baseline systemic inflammation. This differs from the cohort studied by Yan et al., which had higher inflammatory burdens and included bio-experienced patients (13). These baseline differences may lead to low variation in peripheral blood markers. This makes effective risk stratification difficult. Therefore, these findings should not be extrapolated to all UC patients. In bio-naïve patients with a low systemic inflammatory burden, these markers lack utility as standalone predictors of long-term clinical outcomes. Therefore, clinicians should not rely solely on peripheral blood scores, but rather incorporate endoscopic findings or fecal calprotectin.

In contrast, disease duration showed significant independent predictive value in this study. Shorter disease duration significantly correlated with better long-term remission. Currently, the benefit of early biologic intervention in UC remains controversial, which differs from the established “window of opportunity” in Crohn's disease (35). However, our results provide new supportive evidence for early intervention in UC. Persistent inflammation can drive irreversible immune remodeling, forming “immunological scars” (36). This suggests that initiating vedolizumab in the bio-naive stage, before such complex remodeling occurs, may be more critical than considering baseline inflammatory markers alone. This finding provides a basis for optimizing the therapeutic positioning of vedolizumab in first-line treatment. Although baseline static markers had limited predictive power, an exploratory analysis of patients with successful treatment de-escalation before week 54 (*n* = 7) showed that the dynamic decline of SII followed a consistent trend with successful de-escalation. This exploratory finding suggests that longitudinal monitoring of the SII trajectory may reflect systemic inflammatory clearance better than baseline levels alone.

Regarding safety, the serious adverse event rate in this study was only 4.3% (all cases were CMV colitis). No deaths or PML cases were observed. This safety profile is consistent with a large real-world meta-analysis (37) and data from elderly populations (38). Due to its gut-selective mechanism, the risk of CMV infection (39) and tuberculosis (40) with vedolizumab is generally lower than with anti-TNF therapy. Asia has a high burden of tuberculosis (41). In this context, our study confirms that standardized screening and preventive intervention for latent tuberculosis infection effectively ensured treatment safety. This conclusion aligns with a large cohort study from Korea (42).

### 4.4 Study limitations

Crucially, this study is inherently limited by the high rate of missing objective markers, specifically follow-up endoscopic evaluations. As the STRIDE-II guidelines have definitively shifted therapeutic targets toward endoscopic healing (16), our primary reliance on clinical remission restricts the definitive assessment of deep remission. In the real-world setting, patients who achieved symptomatic relief frequently refused follow-up colonoscopies due to the burden of invasive bowel preparation and financial constraints. This dynamic introduces substantial selection bias, dictating that our endoscopic results must be interpreted strictly as exploratory and descriptive observations.

Additionally, the sample sizes for specific exploratory sub-analyses, particularly the paired histological evaluation and the treatment de-escalation subgroup, are inherently small. Lacking the statistical power for formal testing, these secondary findings should be viewed as hypothesis-generating rather than confirmatory. Furthermore, the entire cohort consists of biologic-naive patients. While this effectively eliminates confounding from prior biologic exposure, our conclusions cannot be directly extrapolated to biologic-experienced populations, and these findings require validation in larger prospective studies.

Finally, single peripheral blood markers (such as SII, NLR, and PLR) possess inherent limitations. Currently, they remain purely exploratory markers rather than validated therapeutic targets, and thus cannot substitute for FC. Within our cohort, the integration of routine FC testing was precluded by low patient compliance driven by out-of-pocket costs. Exploring local intestinal microenvironment markers is valuable for guiding personalized treatment (43). Previous studies show a complex bidirectional regulation between neutrophils and the gut microbiota, where microbial metabolites, such as short-chain fatty acids and indoles, can directly regulate neutrophil activation (8). Future studies should dynamically monitor gut microbiota evolution in combination with local inflammatory markers (such as FC and MPO). Furthermore, given the inherent limitations of routine retrospective pathological examinations, future prospective cohorts should incorporate deep profiling techniques, such as immune-confocal microscopy, to explore the microscopic mechanisms underlying VDZ-induced mucosal healing and predict therapeutic responses.

## 5 Conclusion

This study supports the favorable clinical retention, symptomatic relief, and safety of vedolizumab as an initial biologic therapy in Chinese bio-naive UC patients. These results reinforce vedolizumab as an important first-line biologic option for UC patients, particularly highlighting the value of early therapeutic intervention. While baseline blood-count-derived markers showed limited predictive value in this cohort, their dynamic monitoring may offer exploratory references in resource-limited settings. However, given the substantial missing endoscopic data and the reliance on unvalidated exploratory blood biomarkers, our findings regarding mucosal outcomes should be interpreted with caution. Future large-scale prospective studies incorporating mandatory endoscopic evaluation and routine fecal calprotectin monitoring are required to firmly establish these real-world observations.

## Data Availability

The original contributions presented in the study are included in the article/Supplementary material, further inquiries can be directed to the corresponding authors.
