# Supplementary material for: Vedolizumab in biologic-naïve ulcerative colitis: a multicenter real-world evaluation of clinical benefits and exploratory inflammatory indices
Source: Front Med (Lausanne). 2026 Jul 17;13:1897457. doi: 10.3389/fmed.2026.1897457 (PMC13423702; doi:10.3389/fmed.2026.1897457)
Supplement: Supplementary file 1 [file Table_1.docx]

Supplementary Material


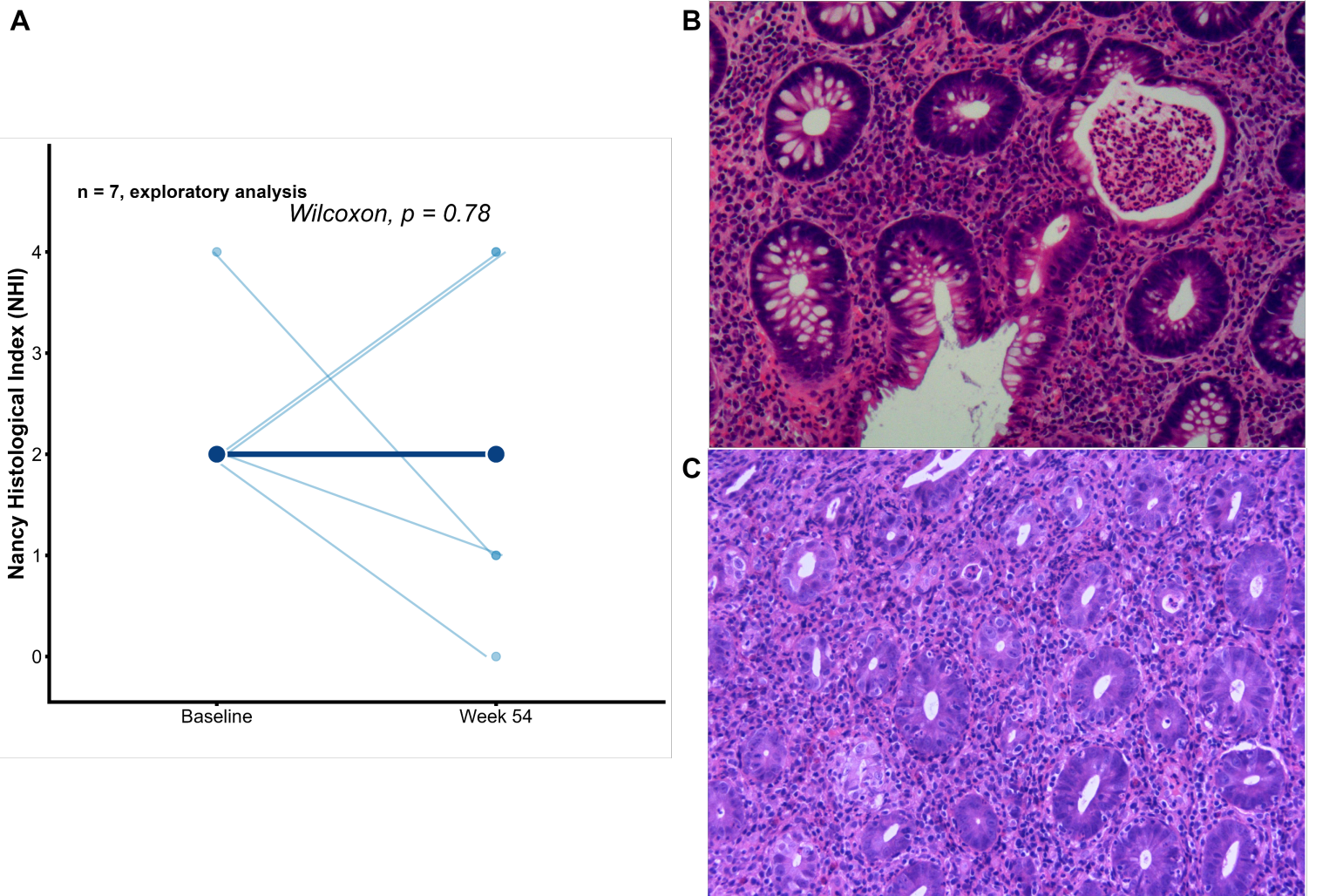


**Supplementary Figure 1.** Histological evaluation at Week 54.

(A) Changes in the Nancy Histological Index (NHI) from baseline to Week 54 in the paired subgroup (n = 7). Light blue lines represent individual changes, and the dark blue line indicates the median. Statistical significance was determined using the Wilcoxon signed-rank test. (B, C) Representative hematoxylin and eosin (H&E)-stained mucosal sections from the same patient at baseline (B) and after 54 weeks of vedolizumab treatment (C).

NHI, Nancy Histological Index; H&E, hematoxylin and eosin.

**Supplementary Table 1:** Granular clinical profiles and rationales for the treatment de-escalation subgroup.

| Patient ID | Timing of De-escalation | Baseline MMS | Baseline MES | MMS at De-escalation | MES at De-escalation | Primary Rationale for Vedolizumab Cessation | Clinical Status at Week 54 |
| --- | --- | --- | --- | --- | --- | --- | --- |
| 1 | Week 48 | 6 | 2 | 1 | 1 | Financial constraints | Clinical Relapse |
| 2 | Week 40 | 5 | 2 | 0 | 0 | preference for oral medications | Maintained Clinical Remission |
| 3 | Week 48 | 5 | 2 | 1 | 0 | Financial constraints | Maintained Clinical Remission |
| 4 | Week 48 | 6 | 2 | 1 | 1 | Financial constraints | Clinical Relapse |
| 5 | Week 32 | 7 | 2 | 2 | 1 | Financial constraints & long-distance commuting | Lost to Follow-up |
| 6 | Week 32 | 5 | 2 | 0 | 0 | Financial constraints | Lost to Follow-up |
| 7 | Week 32 | 6 | 2 | 1 | 1 | Financial constraints | Lost to Follow-up |
| Abbreviations: MMS, Modified Mayo Score; MES, Mayo Endoscopic Subscore. | | | | | | | |
| Note: Any patient experiencing clinical relapse or lost to follow-up prior to Week 54 was strictly classified as a treatment failure in the primary Non-Responder Imputation (NRI) sensitivity analysis. | | | | | | | |
|  | | | | | | | |
